# Body Surface Mapping of Ventricular Repolarization Heterogeneity: An *Ex-vivo* Multiparameter Study

**DOI:** 10.3389/fphys.2020.00933

**Published:** 2020-08-13

**Authors:** Marianna Meo, Pietro Bonizzi, Laura R. Bear, Matthijs Cluitmans, Emma Abell, Michel Haïssaguerre, Olivier Bernus, Rémi Dubois

**Affiliations:** ^1^Institute of Electrophysiology and Heart Modeling (IHU Liryc), Foundation Bordeaux University, Pessac-Bordeaux, France; ^2^University of Bordeaux, CRCTB, Bordeaux, France; ^3^INSERM, CRCTB, U1045, Bordeaux, France; ^4^Department of Data Science and Knowledge Engineering, Maastricht University, Maastricht, Netherlands; ^5^Department of Cardiology, Cardiovascular Research Institute Maastricht, Maastricht University Medical Center, Maastricht, Netherlands; ^6^Bordeaux University Hospital (CHU), Electrophysiology and Ablation Unit, Pessac, France

**Keywords:** sudden cardiac death, ventricular repolarization heterogeneity, body surface potential mapping, T-wave, electrocardiology

## Abstract

**Background:**

Increased heterogeneity of ventricular repolarization is associated with life-threatening arrhythmia and sudden cardiac death (SCD). T-wave analysis through body surface potential mapping (BSPM) is a promising tool for risk stratification, but the clinical effectiveness of current electrocardiographic indices is still unclear, with limited experimental validation. This study aims to investigate performance of non-invasive state-of-the-art and novel T-wave markers for repolarization dispersion in an *ex vivo* model.

**Methods:**

Langendorff-perfused pig hearts (*N* = 7) were suspended in a human-shaped 256-electrode torso tank. Tank potentials were recorded during sinus rhythm before and after introducing repolarization inhomogeneities through local perfusion with dofetilide and/or pinacidil. Drug-induced repolarization gradients were investigated from BSPMs at different experiment phases. Dispersion of electrical recovery was quantified by duration parameters, i.e., the time interval between the peak and the offset of T-wave (T_PEAK_-T_END_) and QT interval, and variability over time and electrodes was also assessed. The degree of T-wave symmetry to the peak was quantified by the ratio between the terminal and initial portions of T-wave area (*Asy*). Morphological variability between left and right BSPM electrodes was measured by dynamic time warping (DTW). Finally, T-wave organization was assessed by the complexity of repolarization index (CR), i.e., the amount of energy non-preserved by the dominant eigenvector computed by principal component analysis (PCA), and the error between each multilead T-wave and its 3D PCA approximation (NMSE). Body surface indices were compared with global measures of epicardial dispersion of repolarization, and with local gradients between adjacent ventricular sites.

**Results:**

After drug intervention, both regional and global repolarization heterogeneity were significantly enhanced. On the body surface, T_PEAK_-T_END_ was significantly prolonged and less stable in time in all experiments, while QT interval showed higher variability across the interventions in terms of duration and spatial dispersion. The rising slope of the repolarization profile was steeper, and T-waves were more asymmetric than at baseline. Interventricular shape dissimilarity was enhanced by repolarization gradients according to DTW. Organized T-wave patterns were associated with abnormal repolarization, and they were properly described by the first principal components.

**Conclusion:**

Repolarization heterogeneity significantly affects T-wave properties, and can be non-invasively captured by BSPM-based metrics.

## Introduction

Abnormal heterogeneity of ventricular repolarization predisposes to the development of life−threatening ventricular arrhythmias and it is associated with increased mortality in the general population (Antzelevitch, 2007; Prenner et al., 2016).

Global ventricular repolarization dispersion is mainly determined by the heterogeneity of action potential durations (APDs) between different myocardial regions, which can be apical-basal (from apex to base of ventricles), transmural (from endocardium to epicardium) and/or interventricular (left vs. right ventricle), and thus affects T-wave properties from surface electrocardiogram (ECG) (Merri et al., 1989; Vinzio Maggio et al., 2012). Classically, global dispersion of repolarization has been defined as the difference between longest and shortest repolarization time (RT) within a set of measurements at multiple sites without regard to their location (Burton and Cobbe, 2001). On the other hand, regional dispersion of repolarization strongly affects T-wave profile as well, therefore measures of local inhomogeneity have also been introduced to take into account spatial distribution of RTs and their variations across adjacent tissue sites. Patients with long QT syndrome display regions with steep repolarization dispersion caused by localized prolongation of APD and genotype-specific alterations in T- wave morphology (Vijayakumar et al., 2014). In Brugada syndrome, sharp local gradients of repolarization and slow conduction areas can both contribute to increased susceptibility to sustained reentrant excitation and are accompanied by T-wave inversion on the body surface (Zhang et al., 2015).

Both abnormal global and local dispersion of repolarization significantly affect T-wave characteristics. Nevertheless, it is still unclear if T-wave properties are more influenced by one of these two conditions, or if both equally contribute to the electrocardiographic alterations of ventricular recovery. Accordingly, the performance of state-of-the-art ECG markers of T-wave has been questioned in clinics, due to their inability to identify vulnerable substrates and reliably distinguish between healthy and diseased subjects (Malik et al., 2000; Van Huysduynen et al., 2005; Smetana et al., 2011; Marill et al., 2018). Since each ECG electrode summarizes the integrated electrical activity over the entire heart, spatial properties of the cardiac generator are lost, and it was shown that features derived from such a low number of electrodes are unlikely to reflect regional changes in myocardial repolarization (Burnes et al., 2001).

To compensate this intrinsic limitation of ECG, high-density body surface mapping (BSPM) has emerged as a promising tool for the diagnosis of T-wave alterations and risk stratification for sudden cardiac death (SCD) (Taccardi et al., 2007; Korhonen et al., 2009). Several studies have confirmed the ability of this technique to capture non-dipolarities of cardiac electrical activity, and on top of that ECG imaging could localize sites of abnormal recovery (Peeters et al., 1998; Burnes et al., 2001; Vijayakumar et al., 2014; Zhang et al., 2015).

Despite these advances, it is still unclear how regional and global repolarization disparities reflect on body surface, and to which extent they can be inferred from BSPMs, as pre-clinical validation is limited and different invasive metrics are used for validation (Fuller et al., 2000; Burnes et al., 2001).

Our study takes a step further from this background and aims to assess the ability of several state-of-the-art and novel BSPM metrics to describe abnormal dispersion of repolarization in an *ex vivo* torso tank model, for which information about actual heterogeneity of repolarization at the heart level is available, and can be used for validation of body-surface indices.

## Materials and Methods

### Experimental Setup and Database

The experimental protocol was approved by Directive 2010/63/EU of the European Parliament on the protection of animals used for scientific purposes and the local ethical committee. Hearts were excised from pigs (*N* = 7, 30–40 kg) and perfused in Langendorff setup as previously reported (Bear et al., 2019a, c). The left anterior descending (LAD) artery was cannulated on a separate perfusion. A 108-electrode sock was attached to the free wall epicardial surfaces of the ventricles. After instrumentation, the heart was suspended in a human-shaped 256-electrode torso tank filled with a Tyrode’s solution. In 3 hearts, pinacidil (Pin) was injected in increasing concentrations (full dose: 30 μM) into the LAD to locally shorten APD and induce gradients in repolarization with other myocardial regions (Pin Group). In the other 4 hearts, Pin administration with comparable doses was preceded by progressive perfusion of non-LAD coronaries with dofetilide (Dof, full dose: 250 nM) to further enhance regional repolarization heterogeneities (early repolarization in the Pin-area, late in the Dof-area). This second set was referred to as Dof & Pin Group. Only experiments presenting all mentioned phases were assigned to one of these categories, therefore other phases which were not common to all experiments (e.g., drug washout) were not included into our analysis. When more recordings were available at baseline, the first recording was included into our analysis. During drug perfusion, the last recording was generally preferred, in order to have enough time for the drug to make effect.

### Signal Preprocessing and Fiducial Point Detection

BSPMs and sock electrograms (EGMs) were simultaneously recorded (BioSemi, Netherlands) at a sampling rate of 2048 Hz during sinus rhythm before and after each drug intervention over *N_*b*_* = 10 beats.

**FIGURE 1:**
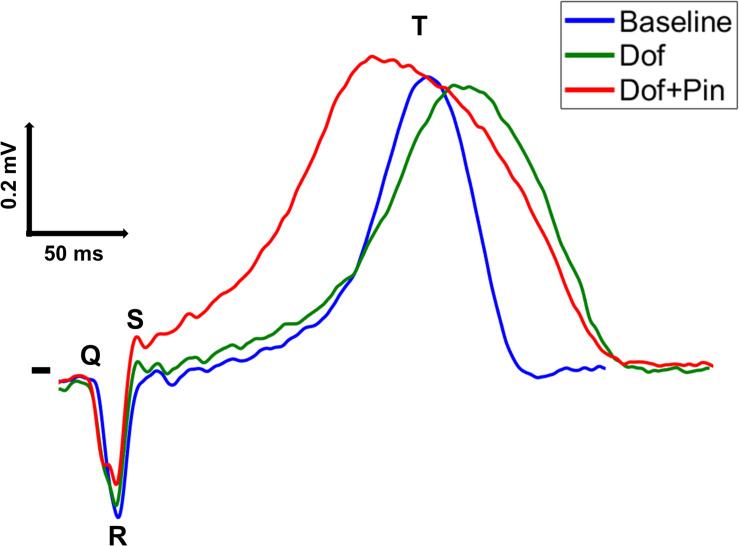
A representative single-lead QRST sequence from a tank potential recorded from an experiment from Dof & Pin Group before (Baseline, blue trace) and after induction of repolarization gradients through injection of dofetilide (Dof, green trace) and simultaneous perfusion with dofetilide and pinacidil (Dof + Pin, red trace).

At each beat *i*, *i* = 1,…,*N*_b_, T-waves from all electrodes were arranged as a *L*×*N* matrix **Y**^(*i*)^ = [**y**(1)…**y**(*N*)] ∈ ℜ^L×N^, with *L=256* equal to the number of tank electrodes, and *N* to the number of time samples. Since electrodes presenting signals with too high levels of noise and/or low voltage on visual inspection have not been included into our analysis, in some cases we may have *L* < 256. A representative single-lead beat with its characteristic deflections from an experiment from Dof & Pin Group is shown at each drug state in Figure 1.

High frequency noise was removed from BSPMs by a low-pass least-squares linear-phase finite impulse response (FIR) filter with cutoff frequency at 40 Hz. Power line interference was suppressed by a discrete Fourier transform-based notch filter. Prior to morphology analysis in section 2.3.3 and 2.3.4, a third-order Savitzky-Golay FIR filter with 50 ms frame length was applied to remove residual noise interferences and further smoothen T-wave profile.

R-wave peak time occurrences were identified from tank potentials as signal local maxima above a preset threshold equal to the half of the signal maximum amplitude value, and at a minimum time distance of 330 ms, using the MATLAB function *findpeaks*. QRS complex onset and offset were detected using a modified version of the algorithm proposed in Nygårds and Sörnmo (1983). Briefly, the envelope of the input signal was computed as the modulus of its Hilbert transform and denoted *x*_env_. In the 60 ms interval preceding each heartbeat, potential candidate QRS onset values ${\tilde{Q\,R\,S}}_{\text{ON}}$ were identified as the local maxima above a fixed threshold equal to 0.2 $\times m\,a\,x\,\left(x_{\text{env}}^{\prime }\right)$, with $m\,a\,x\,\left(x_{\text{env}}^{\prime }\right)$ standing for the maximum value of the numerical gradient of *x*_ev_. In this time frame, we computed the curvature of function *x*_env_ as: Cenv=xenv″1+xenv2′3, with $x_{\text{env}}^{{}^{\prime \prime }}$ equal to the gradient of $x_{\text{env}}^{\prime }$. Doing so, the exact time location of each QRS onset QRS_ON_ could be finally determined as that of *C*_env_ local maximum in the 100 ms interval before the earliest ${\tilde{Q\,R\,S}}_{\text{ON}}$.

An improved version of Woody’s method for time delay estimation was applied to each BSPM electrode to detect the offset of T-wave (T_OFF_) (Cabasson and Meste, 2008). This method introduces an iterative maximum likelihood estimator to analyze variable latency signal. According to this approach, at each instant *t* a generic signal *x*_i_(*t*) can be modeled as:

$$\,x_{i}\,\left(t\right)=s\,\left(t-d_{i}\right)+e_{i}\,\left(t\right),$$

with *s*(*t*−*d*_i_) defined as the unknown reference wave delayed by *d*_i_ and *e*_i_(*t*) as observation’s white Gaussian noise, for i = 1,…,*N*_b_. For our application, a fixed-length search window has been used for all electrodes to detect the end of T-wave at each heartbeat, with duration set between 200 and 300 ms depending on signal characteristics. As demonstrated by Cabasson and Meste (2008), each T-wave can be detected under an iterative scheme which determines the time lags ${\hat{d}}_{\text{i}},i=1,\ldots ,N_{\text{b}}$ as those maximizing the correlation with a template waveform:

$${\hat{d}}_{i}=\arg m\,a\,x_{d}\,\left[\sum_{t}\sum_{i}^{N_{b}}\sum_{k>i}^{N_{b}}x_{k}\,\left(t+d_{k}\right)\,x_{i}\,\left(t+d_{i}\right)\right]$$

Under the constraint that $\sum_{\text{i}}d_{\text{i}}=0$, and with the template wave equal to the mean of all delayed waves *x*_k_(*t* + *d*_k_) except for the one currently examined. The delays ${\hat{d}}_{\text{i}}$ are finally used to determine the final location of T-wave end T_OFF_ after each R-wave. The algorithm was applied to each electrode separately, but with a fixed search frame.

T-wave peak (T_PEAK_) was detected from each lead as the time instant of the local maximum (minimum) of T-wave with positive (negative) polarity in the time interval starting 100 ms after the R-wave occurrence and ending at T_END_. The onset of T-wave (T_ON_) was finally identified at 0.3×T_FRAME_ samples after R-wave peak time instant, with T_FRAME_ equal to the median duration of the R-peak-T-wave offset interval over all leads. The time location of T-wave fiducial points was corrected if possible, otherwise BSPM electrodes associated with misdetections due to flat repolarization were discarded from our analysis.

### Standard and Novel Electrocardiographic Features of Myocardial Repolarization

In this section, we introduce several descriptors of T-wave and we evaluate their ability to non-invasively capture abnormal gradients in repolarization. We investigate to which extent the heterogeneity observed at the level of the tissue can be quantified on body surface, as abnormal dispersion of repolarization is known to strongly affect T-wave characteristics, in terms of: (a) duration; (b) variability duration; (c) shape; (d) spatial complexity, although the mechanistic link is not always clear and findings from previous studies are sometimes in mutual contradiction (Priori et al., 1997; Antzelevitch, 2001; Porthan et al., 2013). Accordingly, moving from previous research we define some markers of duration, morphology and spatial content of T-wave. Since in previous works most of these indices have been merely assessed from one or more beats in single-lead or 12-lead ECGs, we investigated whether equivalent BSPM-derived metrics could provide equally or more detailed information on repolarization abnormalities in our torso-tank model. A brief definition of each BSPM- and EGM-based indices is presented in Table 1.

**TABLE 1:** Summary of all EGM- and BSPM-derived metrics of ventricular repolarization investigated in this study.

	Repolarization property	Definition
**BSPM index**		
T_PEAK_-T_END_ (ms)	Duration	Median duration of the peak-to-end portion of T-wave per BSPM lead (over heartbeats)
QT_C_ (ms)	Duration	Median duration of the QT interval corrected for the heart rate (Bazett’s formula) per BSPM lead (over heartbeats)
SD(T_PEAK_-T_END_) (ms)	Variability of duration (temporal)	Standard deviation of the peak-to-end portion of T-wave per BSPM lead (over heartbeats)
QTD (ms)	Variability of duration (spatial)	Difference between the maximum and the minimum QT_C_ value at each heartbeat
*Asy* (a.u.)	Morphology	Median value of the ratio between of the areas of the peak-to-end portion and that between the onset and the peak of T-wave (over heartbeats)
*d*_LV/RV_ (mV)	Morphology	Cumulative DTW distance between all the possible pairs of electrodes from the right and left sides of the torso tank (associated with the RV and LV, respectively)
CR (%)	Spatial complexity	Ratio of the mean square of the second through *L*-th eigenvalues to the root mean square of all eigenvalues multiplied by 100
NMSE (%)	Spatial complexity	Average (over electrodes and beats) of the normalized mean square errors between the multilead T-wave at a given heartbeat and its PCA projection onto a 3D subspace from the T-wave at a different heartbeat
**EGM index**		
RTG_GLOBAL_ (ms)	RT global epicardial dispersion	Absolute difference of the average RTs over sock electrodes from differently perfused epicardial sites (Pin Group: Pin vs. no-Pin; Dof & Pin Group: Dof vs. Pin)
RTG(LV/RV)_GLOBAL_ (ms)	RT global interventricular epicardial dispersion	Absolute difference of the average RTs over sock electrodes from differently perfused epicardial sites (Pin Group: Pin vs. no-Pin; Dof & Pin Group: Dof vs. Pin)
RTG_LOCAL_ (ms/mm)	RT local epicardial dispersion	Temporal average of the maximum absolute differences between RTs from two adjacent epicardial sites, divided by their Euclidean distance

#### Duration (T_PEAK_-T_END_, QT_C_)

Time domain descriptors of ventricular electrical recovery were computed from surface potentials at each experimental stage. At each beat and from each electrode, we measured the duration of the time interval between the peak T_PEAK_ and the end of T-wave T_OFF_, which has been regarded as an index of transmural dispersion of repolarization (Yan and Antzelevitch, 1998; Antzelevitch et al., 1999). For our analysis, the median value of the duration of this interval over all heartbeats was computed from each electrode and denoted T_PEAK_-T_END_.

We also investigated the role of QT interval as a marker for impaired electrical recovery, as its abnormal prolongation is known to be proarrhythmic (Straus et al., 2006). Accordingly, at each beat *i* and in each electrode ℓ the duration QT_ℓ,*i*_ of the interval between the beginning of the QRS complex Q_ON_ and the T-wave offset T_OFF_ was measured and corrected for heart rate using Bazett’s formula QT_C_ℓ_,i_ = QT_ℓ,*i*_/(RRm)^0.5^, with RRm equal to the mean RR interval. The median of corrected QT values over all beats was computed from each tank electrode (QT_C_) and used for subsequent analysis.

#### Duration Variability [SD(T_PEAK_-T_END_), QTD]

The variability of cardiac repolarization was also determined in terms of temporal or beat-to-beat variability. Accordingly, we assessed temporal inhomogeneity of T_PEAK_-T_END_ duration by computing the standard deviation of the peak-to-end T-wave interval durations across heartbeats from each BSPM electrode and denoted SD(T_PEAK_-T_END_), as it is known to be increased in subjects at higher risk for SCD (Piccirillo et al., 2013).

Finally, QTc interval dispersion (QTD) was also calculated as the difference between maximum and minimum QT over BSPM electrodes, being a routine measure of spatial heterogeneity of repolarization in clinics (Baumert et al., 2016).

#### Shape (*Asy*, *d_LV/RV_*)

As in Di Bernardo and Murray (2000) and Sheridan et al. (2010), in each electrode ℓ = 1,…,*L* and at each heartbeat beat *i*, *i* = 1,…,*N*_b_, we computed an index of symmetry of T-wave as the ratio between the area of the terminal portion of the repolarization (corresponding with the T_PEAK_-T_END_ interval) and that between the onset and the peak of T-wave (i.e., the T_ON_-T_PEAK_ interval):

$$A\,s\,y_{\ell ,i}=\frac{\int_{T_{P\,E\,A\,K}}^{T_{E\,N\,D}}y_{\ell ,i}\,\left(t\right)\,d\,t}{\int_{T_{O\,N}}^{T_{P\,E\,A\,K}}y_{\ell ,i}\,\left(t\right)\,d\,t}$$

According to these works, more symmetrical T-waves were associated with increased repolarization heterogeneity, with index values closer to unity. Indeed, normal T waves should have a gradual upstroke with a slightly steeper descending limb, and abnormal dispersion of repolarization seems to alter relative contribution of these two portions. In our study, T-waves were mean-centered and normalized between 0 and 1. Integrals were discretized through the trapezoidal rule. The median of the asymmetry index over all beats was determined from each BSPM electrode and taken as a marker of repolarization dispersion (*Asy*).

T-wave morphology aberrations have been linked to abnormal heterogeneity of ventricular repolarization and increased arrhythmia vulnerability. Right-to-left ventricle dispersion is an important factor contributing to heterogeneous repolarization both in physiological (Hlaing et al., 2005) and pathological conditions (Fish et al., 2004), and it can affect T wave profile (Srinivasan et al., 2019). Dynamic time warping (DTW) has been previously proposed to describe T-wave beat-to-beat morphological changes, assess temporal variability of ventricular recovery from 12-lead ECG and predict SCD risk in chronic heart failure patients (Ramirez et al., 2017). Accordingly, moving from this theory we investigated whether drug-induced repolarization gradients may contribute to interventricular dispersion and be assessed by T-wave shape analysis. Specifically, we split BSPM electrodes into a set of *L*_*LV*_ electrodes associated with the left ventricle (LV; from the left side of the heart) and one consisting of *L*_*RV*_ electrodes for the right ventricle (RV; from the right side of the heart), and we applied DTW to measure the degree of similarity between waveforms from pairs of tank potentials, each associated with a different ventricle. Algorithm details are reported elsewhere (Müller, 2007). Briefly, this approach aims to find the match between two temporal sequences along an optimal alignment path by minimizing a cost function. In our study we choose the Euclidean distance as a similarity metric. A median T-wave signal was computed over all beats from each electrode and given as input to the algorithm after mean-centering and signal normalization by L2-norm. The cumulative distance *d*_LV/RV_ minimizing the warping path between the examined pair of signals was used as a T-wave parameter, with higher values of the index denoting higher dissimilarity between the waveforms and increased heterogeneity of the ventricular recovery.

#### Complexity (CR, NMSE)

Due to its ability to capture inhomogeneities and variability of repolarization, principal component analysis (PCA) has been suggested as a tool to measure the spatial organization of T-wave (Priori et al., 1997; Malik et al., 2000; Zabel and Malik, 2001), under the hypothesis that cardiac activity can be approximated to a 3D electric dipole (Holt et al., 1969), and that components outside the T-wave loops would reflect pathological inhomogeneities and represent an arrhythmic substrate (Okin et al., 2002; Porthan et al., 2013). Accordingly, in line with this research, we assessed body surface electrical organization as follows. At each heartbeat *i*,*i* = 1,…,*N*_b_, a multi-electrode T-wave matrix **Y**^(i)^,*i* = 1,…,*N*_b_ was segmented between T_ON_ and the median value of T_END_ over all leads. T-wave matrix was then factorized by singular value decomposition **Y**^(i)^ = **UΣV**^T^, with ***U*** and ***V*** representing the right and left singular vectors, respectively, and **Σ** being a diagonal matrix containing the singular values σ_ℓ_, ℓ = 1,…,*L*. As in Priori et al. (1997), we measured the relative contribution of the dominant eigenvector over total signal variation by computing the complexity of repolarization (CR) as:

$$C\,R=\sqrt{\frac{\sum_{\ell =2}^{L}\sigma_{\ell }^{2}}{\sum_{\ell =1}^{L}\sigma_{\ell }^{2}}}\,100$$

Lower CR values were assumed to describe less complex T-wave patterns and homogeneous repolarization, as one major spatial component (eigenvector) should be able to accurately describe such an organized process. Conversely, spatial repolarization heterogeneities were expected to require a higher number of components for accurate PCA reconstruction, thus yielding higher CR.

Additionally, we used PCA to explore the temporal variability of T-wave spatial components over higher-order spaces. Specifically, after normalization between 0 and 1 and mean-centering, at each beat, *i* = 1,…,*N*_b_ PCA was applied to **Y**^(i)^ to compute a 3D subspace **M**^(*i*)^ onto which every subsequent T-wave**Y**^(j)^,*j*≠*i*, was projected. The normalized mean square error (NMSE) between the input potentials **Y**^(j)^ and their low-rank PCA approximation was then computed as:

$$N\,M\,S\,E^{\left(j\right)}=\frac{\left(\text{I}-{\text{M}}^{\left(i\right)}\,{\text{M}}^{\left(i\right)\,\#}\right)\,{\text{Y}}^{\left(\text{j}\right)}}{{\text{Y}}^{\left(\text{j}\right)}}\,100$$

For *j* = 1,…,*N*_b_, *j*≠*i*, with **M**^(*i*)#^ = [**M**^(*i*)T^**M**^(*i*)^]^−1^**M**^(*i*)T^ being the pseudo-inverse of **M**^(*i*)^ (Bonizzi et al., 2010; Meo et al., 2013). At each beat, the average NMSE over all tank electrodes was finally considered as a descriptor of T-wave repolarization, assuming that higher reconstruction error would render a higher degree of spatiotemporal dispersion of repolarization.

### Validation of Body Surface Signal Processing Algorithms

To confirm whether changes in BSPM parameters across each experiment truly reflected alterations in repolarization characteristics, at each experimental step every non-invasive index was averaged over all electrodes and/or heartbeats, and their time evolution across the experiment was compared with that of several invasive epicardial markers of repolarization dispersion.

To verify the ability of BSPM-derived features to assess global dispersion of repolarization, at each heartbeat we computed the absolute difference RTG_GLOBAL_ between the spatial average of RTs over sock electrodes from differently perfused epicardial regions, i.e., between mean RT from electrodes in the Pin-area and those in non-perfused sites for Pin Group, and between mean RT from electrodes in the Pin-area and those in the Dof-area for Dof & Pin Group.

Additionally, at each beat we also measured the absolute difference between mean RTs from sock electrodes in contact with LV and those attached to RV (RTG(LV/RV)_GLOBAL_).

We also investigated whether body surface analysis could identify regional repolarization gradients at a smaller scale. Accordingly, for validation at each beat we quantified the local spatial RT gradient RTG_LOCAL_, i.e., the temporal average of the maximum differences between RTs from two adjacent EGM sites, divided by their Euclidean distance (Vijayakumar et al., 2014). The higher its value, the steeper the local repolarization gradients between neighboring sites. Specifically, for experiments from Pin Group, each invasive and non-invasive parameter was assessed: (1) at the beginning of the experiment, in the absence of drug-induced repolarization gradients (“Baseline”); (2) during LAD coronary perfusion with half concentration of pinacidil (15 μM, “1/2 Pin”); (3) during perfusion of the same site will full concentration of pinacidil (30 μM, “1/1 Pin”). Similarly, we computed all parameters for Dof & Pin Group at three experimental phases as well: (1) “Baseline”; (2) during injection of dofetilide (250 nM) in extra-LAD areas (“Dof”); (3) during injection of pinacidil (between 15 and 30 μM) in the LAD coronary (“Dof + Pin”). While in the Pin Group it has been possible to inject the total drug dose during all interventions, in two hearts (#3 and #4) from the Dof & Pin Group the experiments could not be completed due to some technical problems with LAD cannulation, thus only half dose of pinacidil could be used. Nevertheless, a clear effect of the drug on these additional hearts was observed despite the use of a lower pinacidil concentration, with visible and interpretable impact on repolarization properties, thus all the signal recordings could be eventually used and analyzed. To better understand the ability of each parameter to capture changes in each experimental phase from baseline, we also examined the trend of differences between the value of each feature at a specific experimental stage and that measured at baseline. For Pin Group, interphase variations were referred to as “Δ1/2 Pin” and “Δ1/1 Pin,” while for Dof & Pin Group these were labeled as “ΔDof” and “ΔDof + Pin.” Variations in body surface parameters were then compared to those in invasive measures of repolarization.

Finally, to verify the agreement of each non-invasive parameter with EGM gradients of repolarization, a Bland-Altman (BA) plot for each BSPM index from all experimental phases was built as follows (Altman and Bland, 1999). After feature standardization, the difference between the examined T-wave marker and the reference invasive metric z_D_ was displayed against their average z_M_. The mean bias was estimated as the average of all differences z_D_, to provide an estimate of the average discrepancy between methods. For the same purpose, we also determined the 95% limits of agreement (LoA) of the interval as a measure of confidence. The narrower the interval, and the higher the number of points within it, the higher the degree of agreement between the compared approaches. Confidence bounds were corrected for repeated measures’ data, assuming that the true difference value is not constant across observations as in Altman and Bland (1999).

## Results

### Pin Group

**FIGURE 2:**
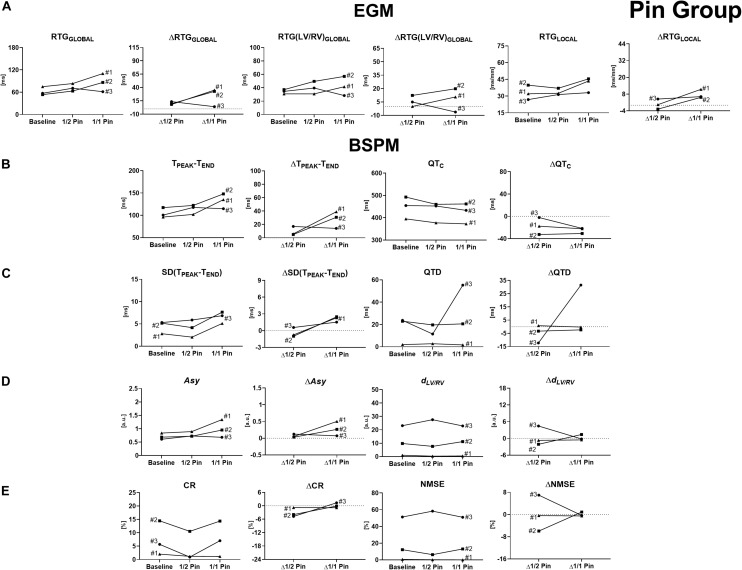
Time evolution of signal features and their variations with respect to baseline conditions for experiments from Pin Group, computed before and during progressive injection with pinacidil. **(A)** Invasive epicardial metrics of heterogeneity of repolarization and changes from baseline computed as global differences in RTs between Pin- and not-Pin sites (RTG_GLOBAL_ and ΔRTG_GLOBAL_, left), global differences in RTs between LV and RV (RTG(LV/RV)_GLOBAL_ and ΔRTG(LV/RV)_GLOBAL_, middle), and local RT gradients between neighboring epicardial sock nodes (RTG_LOCAL_ and ΔRTG_LOCAL_, right). **(B)** BSPM indices and changes from baseline of T-wave duration (T_PEAK_-T_END_ and ΔT_PEAK_-T_END_, left) and corrected QT interval (QT_C_ and ΔQT_C_, right). **(C)** BSPM indices and changes from baseline of temporal variability of T-wave duration [SD(T_PEAK_-T_END_) and ΔSD(T_PEAK_-T_END_), left] and QT_C_ spatial dispersion (QTD and ΔQTD, right). **(D)** BSPM indices and changes from baseline of T-wave symmetry (*Asy* and Δ*Asy*, left) and shape (*d*_LV/RV_ and Δ*d*_LV/RV_, right). **(E)** BSPM indices and changes from baseline of T-wave complexity (CR and ΔCR, left, and NMSE and ΔNMSE, right). a.u., arbitrary units.

Time evolution of EGM- and BSPM-derived parameters for each experiment from Pin Group is shown in Figure 2.

More pronounced differences in electrical recovery timing between perfused and non-perfused regions are globally measured by an increase in RTG_GLOBAL_ in all experiments, except for experiment #3 (Figure 2A, left). Pinacidil injection is also responsible for a marked increase in EGM interventricular RT dispersion RTG(LV/RV)_GLOBAL_ in experiments #1 and #2 (Figure 2A, middle), whereas RTs become more similar with time in experiment #3. Similar dynamics can be locally retrieved, with higher regional dispersion of repolarization and higher RTG_LOCAL_ in all experiments #1 and #2 during drug injection, although with a more blunted evolution in experiment #3.

As shown in Figure 2B (left), the evolution of T_PEAK_-T_END_ on body surface is consistent with the trend of all epicardial measures of dispersion, namely, an increase in the duration of the ending portion of T-wave can be observed when repolarization is more heterogeneous. Regarding experiment #3, T_PEAK_-T_END_ prolongation is more pronounced when pinacidil injection has started, and then a drop in the index can be observed, with a time evolution resembling more that of global invasive markers of repolarization dispersion (RTG_GLOBAL_ and RTG(LV/RV)_GLOBAL_) rather than regional measures (RTG_LOCAL_). On the other hand, a progressive shortening of QT_C_ duration is observed during pinacidil perfusion throughout all interventions (Figure 2B, right), a behavior that has been reported by several experimental studies (Padrini et al., 1992; Extramiana and Antzelevitch, 2004), but in opposition to the time evolution of invasive markers of repolarization dispersion (except for RTG_LOCAL_ in experiment #3).

The SD(T_PEAK_-T_END_) index measures increasing temporal variability of T_PEAK_-T_END_ in all experiments (Figure 2C, right), but with transient fluctuations in parameter values in experiments #1 and #2 that do not always reflect the evolution of EGM metrics when low doses of pinacidil are administered. For experiment #3, temporal increase SD(T_PEAK_-T_END_) does not match the decreasing trend of global markers of epicardial dispersion, while it better captures changes in local recovery heterogeneity RTG_LOCAL_. Overall, changes between the beginning and the end of all interventions ΔSD(T_PEAK_-T_END_) are similar to that of the invasive counterparts, thus confirming that enhanced repolarization dispersion at the level of tissue reflects on a less stable temporal distribution of T_PEAK_-T_END_ duration on body surface. Spatial dispersion of QTc (QTD) is overall stable in experiments #1 and #2 (Figure 2C, left), while a marked, unexpected increase in QTD is measured during experiment #3, which is in disagreement with all invasive global metrics of dispersion. The same trends were observed without heart rate correction (results not shown).

As reported in Figure 2D (left), at baseline T-waves are usually left-skewed, and this characteristic is exacerbated by the introduction of global and regional repolarization gradients in experiments #1 and #2. Concerning experiment #3, T-waves also tend to be more left-skewed since the beginning of the intervention and keep a stable shape in time, as global and interventricular RT gradients from EGMs become more homogeneous. Overall, higher heterogeneity of repolarization seems to be associated with more asymmetric T-waves on body surface, in contrast to previous studies (Di Bernardo and Murray, 2000).

Interventricular differences in T-wave morphology between right and left BSPM electrodes according to DTW processing appear overall modest (Figure 2D, right). Drug-induced changes in repolarization properties significantly enhance such differences and lead to an increase in DTW distance *d*_*LV*/*RV*_ only in experiment #2, and this is invasively confirmed by an increase in RT(LV/RV)_G__L__OBAL_ during pinacidil injection, and by the other epicardial metrics as well. On the contrary, during the other interventions the index tends to have a stable trend, and changes between the initial and the final steps of the experiments are limited, in spite of a marked increase in EGM interventricular RT dispersion.

Unlike previous works (Priori et al., 1997), unexpected patterns of electrical organization are revealed by the dominant PCA spatial direction at low doses of pinacidil, with a drop in CR values when starting the perfusion (Figure 2E, left). At the final drug state, CR values are again comparable to those measured at baseline in all hearts. Similarly, body surface analysis of repolarization complexity according to the NMSE parameter in Figure 2E (right) also highlights some variability in its temporal evolution and absolute values across the interventions. In addition, changes in the parameter values from baseline ΔNMSE are modest. Taken together, these findings indicate that the accuracy of T-wave projection onto low-dimension spaces is quite variable and somehow difficult to link with the trend of the reference EGM metrics in this framework.

**FIGURE 3:**
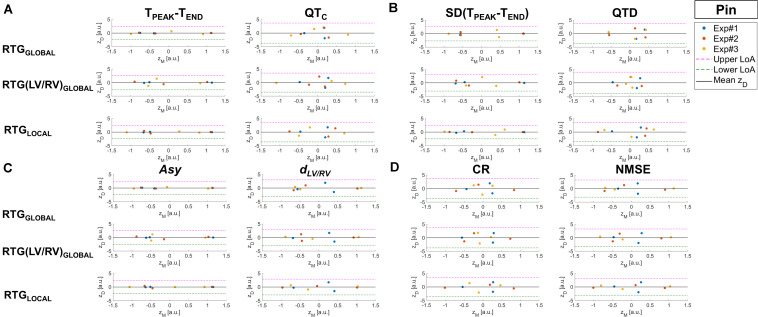
BA plots of the differences z_D_ between BSPM markers of T-wave and invasive RT dispersion measures vs. their mean z_M_ for the assessment of the agreement between the two approaches for experiments from Pin Group before and during progressive injection with pinacidil. The mean difference between methods (horizontal continuous black line) and upper and lower 95% LoA (dashed magenta and green lines, respectively) are also displayed. **(A)** BSPM indices and T-wave duration (T_PEAK_-T_END_, left) and corrected QT interval (QT_C_, right). **(B)** BSPM indices of temporal variability of T-wave duration [SD(T_PEAK_-T_END_), left] and QT_C_ spatial dispersion (QTD, right). **(C)** BSPM indices of T-wave symmetry (*Asy*, left) and shape (*d*_LV/RV_, right). **(D)** BSPM indices of T-wave complexity (CR, left, and NMSE, right). a.u., arbitrary units.

The agreement between T-wave markers and measures of epicardial RT dispersion is assessed by BA plots in Figure 3.

For all T-wave indices, mean difference between non-invasive and invasive analysis (horizontal black line) is close to zero and points are quite scattered above and below zero, suggesting that there is no systematic bias (i.e., the error between BSPM- and EGM-derived parameters z_D_ does not present any specific trend). The T_PEAK_-T_END_ index (Figure 3A, left) is overall highly consistent both with global (RTG_GLOBAL_) and local descriptors (RTG_LOCAL_) of repolarization heterogeneity, as the difference z_D_ between the measures derived from invasive and non-invasive approaches tends to zero and the band of agreement (LoA) is quite narrow. With regard to the analysis of interventricular dispersion [RTG(LV/RV)_GLOBAL_], scatter points associated with experiment #3 tend to depart from the line z_D_ = 0, but they are still within the LoA bounds. BA analysis of QT interval (Figure 3A, right) highlights that points associated with low QT_C_ values (i.e., late experimental stages) are close to zero, regardless of the EGM marker, whereas longer QT_C_ characterize points which lie farther from this threshold, although no specific trend can be elucidated from this distribution. Furthermore, the large width of the confidence interval denotes lower certainty with regard to the relation with epicardial RT dispersion at each scale of observation. In Figure 3B (left) BA plots for SD(T_PEAK_-T_END_) confirm good agreement with all measures of RT gradients, especially with that of global dispersion RTG_GLOBAL_, with z_D_ values nearly equal to zero and well separated point clusters (points associated with experiments #1 and #2 are well separated from those related to experiment #3). Similar remarks can be done for the regional analysis of heterogeneity through RTG_LOCAL_, but with z_D_ values closer to zero for experiment #3, suggesting that in this heart local dispersion of ventricular recovery was better captured by this index than in the global analysis. The ability to distinguish between distinct drug states is lost when considering spatial dispersion of duration QTD (Figure 3B, right), with most of z_D_ points far from zero and a large LoA interval. The relation between T-wave morphology and epicardial RT heterogeneity is displayed in Figure 3C. For the *Asy* index (left panel), BA plot demonstrates accurate agreement with regional and global dispersion of repolarization, and measures acquired from different experiments (i.e., #1 and #2) at the same phase tend to cluster around z_D_ = 0. Measures related to experiment #3 also follow well global EGM metrics, and they are distributed far from each other along z_D_ = 0. Also, the bounds of the interval of agreement are quite narrow. The scatterplots for the *d*_LV/RV_ index in in Figure 3C (right) are more scattered, but at acceptable distance from the zero-threshold and comparable agreement with all EGM parameters. Finally, BA analysis of T-wave complexity according to the CR and NMSE indices (Figure 3D, left and right panel, respectively) denotes even lower agreement with invasive RT dispersion, as confirmed by the quite large LoA interval and z_D_ distribution, with most of the points far from zero.

### Dof & Pin Group

**FIGURE 4:**
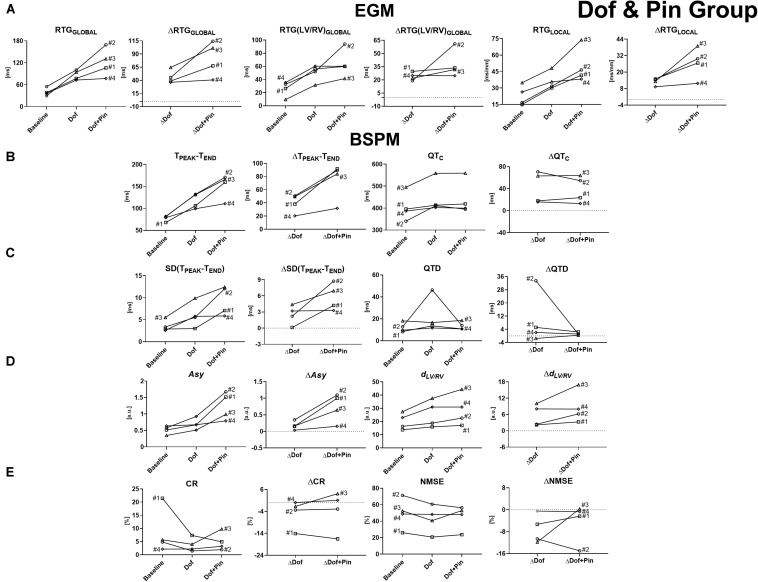
Time evolution of signal features and their variations with respect to baseline conditions for experiments from Dof & Pin Group, computed before and during progressive injection with dofetilide and pinacidil. **(A)** Invasive epicardial metrics of heterogeneity of repolarization and changes from baseline computed as global differences in RTs between Pin- and not-Pin sites (RTG_GLOBAL_ and ΔRTG_GLOBAL_, left), global differences in RTs between LV and RV (RTG(LV/RV)_GLOBAL_ and ΔRTG(LV/RV)_GLOBAL_, middle), and local RT gradients between neighboring epicardial sock nodes (RTG_LOCAL_ and ΔRTG_LOCAL_, right). **(B)** BSPM indices and changes from baseline of T-wave duration (T_PEAK_-T_END_ and ΔT_PEAK_-T_END_, left) and corrected QT interval (QT_C_ and ΔQT_C_, right). **(C)** BSPM indices and changes from baseline of temporal variability of T-wave duration [SD(T_PEAK_-T_END_) and ΔSD(T_PEAK_-T_END_), left] and QT_C_ spatial dispersion (QTD and ΔQTD, right). **(D)** BSPM indices and changes from baseline of T-wave symmetry (*Asy* and Δ*Asy*, left) and shape (*d*_LV/RV_ and Δ*d*_LV/RV_, right). **(E)** BSPM indices and changes from baseline of T-wave complexity (CR and ΔCR, left, and NMSE and ΔNMSE, right). a.u., arbitrary units.

The same analysis has been repeated for all experiments from Dof & Pin Group, and the trend of invasive and non-invasive descriptors of ventricular repolarization is reported in Figure 4.

In all experiments the injection of dofetilide has favored the onset of global and regional electrical disparities, which are further accentuated by the perfusion with pinacidil later in each experiment. These effects are correctly captured by all EGM metrics (Figure 4A), and they are overall stronger than in the setup of Pin Group, due to the combined and opposite effect of the two drugs on the duration of the recovery time.

As for Pin Group, an increasing evolution of T_PEAK_-T_END_ with the course of time also characterizes experiments from Dof & Pin Group and accurately follows changes in repolarization properties as measured at the level of the epicardium (Figure 4B, left). QTc index can also describe electrical recovery alterations (Figure 4B, right), although its variations over time are less abrupt than for T_PEAK_-T_END_ and pinacidil injection seems to have little effect compared with the previous drug state.

Likewise, an increase in temporal variability in the duration of the final portion of T-wave is described by an increase in SD(T_PEAK_-T_END_), which is more evident when both drugs are injected (except for experiment #4, where pinacidil has little effect on the index) (Figure 4C, left). Trends of SD(T_PEAK_-T_END_) and its changes from baseline ΔSD(T_PEAK_-T_END_) closely reflect the appearance of repolarization gradients through pharmacological approach as confirmed by the corresponding evolution of all epicardial measures of RT dispersion with the course of the experiments. Conversely, drug-induced changes in repolarization heterogeneity were not captured by QTD (Figure 4C, right), whose values at the end of the perfusion of both drugs were comparable with those measured at baseline, with and without heart rate normalization, and with high variability in its values across the interventions when only dofetilide is injected.

In this set of interventions, T-wave morphology is significantly affected during drug perfusion. Globally, T-wave shape tends to be more triangular/trapezoidal, the ascending slope is steeper and a notched pattern can be frequently observed from BSPMs (see Figure 1), compared with Pin Group, at baseline T-waves are either left-skewed or symmetric, with average values of *Asy* inferior to unity (Figure 4D, left). Subsequently, late repolarization is prolonged by the injection of dofetilide, thus durations of the starting and final portions are less similar to each other. These differences are further exacerbated by the injection of pinacidil, which drastically shortens the duration of early repolarization and causes a strong increase in *Asy* values. Time evolution of this body surface marker and its changes from baseline closely reflect the trend of the epicardial repolarization dispersion.

Drug-induced repolarization gradients are also responsible for more pronounced differences in T-wave shape between left and right BSPM electrodes according to DTW analysis. As shown in Figure 4D (right), a lower degree of similarity between waveforms from left and right side of the torso tank is assessed by DTW during dofetilide perfusion for all interventions, and it is in keeping with the evolution of all EGM measures of RT dispersion. Interventricular differences in T-wave patterns are further emphasized by the injection of pinacidil later in both experiments, and confirmed by EGM analysis.

As for the previous set of interventions, the evolution of PCA-derived features in Figure 4E is affected by strong inter-experiment variability, although our findings seem to support the idea that overall higher heterogeneity of the ventricular recovery is better described by low-rank approximations of cardiac repolarization. In all experiments, lower PCA reconstruction errors and a sharp decrease in the CR index are measured during dofetilide perfusion. A further (although more modest) drop in this parameter is observed when adding pinacidil, except for experiment #3. According to NMSE analysis, a decline in spatial complexity with time can be easily appreciated for experiment #2, whereas the other ones are characterized by modest changes from baseline and weak agreement with invasive assessment of repolarization gradients.

**FIGURE 5:**
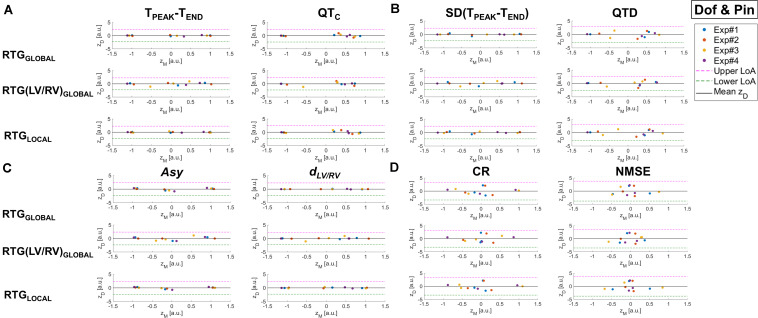
BA plots of the differences z_D_ between BSPM markers of T-wave and invasive RT dispersion measures vs. their mean z_M_ for the assessment of the agreement between the two approaches for experiments from Dof & Pin Group before and during progressive injection with dofetilide and pinacidil. The mean difference between methods (horizontal continuous black line) and upper and lower 95% LoA (dashed magenta and green lines, respectively) are also displayed. **(A)** BSPM indices and T-wave duration (T_PEAK_-T_END_, left) and corrected QT interval (QT_C_, right). **(B)** BSPM indices of temporal variability of T-wave duration [SD(T_PEAK_-T_END_), left] and QT_C_ spatial dispersion (QTD, right). **(C)** BSPM indices of T-wave symmetry (*Asy*, left) and shape (*d*_LV/RV_, right). **(D)** BSPM indices of T-wave complexity (CR, left, and NMSE, right). a.u., arbitrary units.

In Figure 5, BA plots for all BSPM parameters are reported. As for Pin Group, mean bias is negligible for all indices. However, dispersion of point distribution is globally lower than in Pin Group, with z_D_ values close to zero and no specific pattern in relation to z_M_. Specifically, in Figure 5A (left), the relation between T_PEAK_-T_END_ and global and local descriptors of epicardial RT dispersion is displayed. Overall, experimental measures from different phases are even better separated from each other along the z_M_ horizontal axis than in Pin Group, and their variability at a given phase is low, especially when compared to RTG_GLOBAL_ and RTG_LOCAL_. Moreover, the LoA interval is quite narrow for all scatterplots, hinting at good agreement with invasive RT gradient metrics, regardless of the scale of observation. Conversely, with regard to QT interval analysis (Figure 5A, right), while the interval of agreement has similar bounds and baseline z_D_ difference values (left side of the scatterplot) tend to zero, then with progressive drug administration their distribution appears more scattered around the mean bias, and measures acquired at different drug states tend to form a unique cluster, denoting a lack of sensitivity of the QT_C_ index and poor discrimination of different drug states (e.g., different degrees of RT heterogeneity). Similar remarks can be done for the indices of variability of body surface repolarization duration (Figure 5B), with SD(T_PEAK_-T_END_) (left panel) exhibiting closer agreement with all invasive RT gradient parameters at each experimental phase than QTD (right panel). Taken together, these findings suggest that the T-wave final portion is more informative than QT interval with regard to the characterization of distinct levels of repolarization heterogeneity. BA plots for T-wave shape parameters are shown in Figure 5C. T-wave asymmetry (left panel) and interventricular DTW distance (right panel) are both more consistent with EGM reference indices than their counterparts from Pin Group, as denoted by all quasi-null z_D_ values, and confidence intervals are quite tight. Of note, *Asy* values from the same experimental phase tend to form definite clusters, thus denoting lower measure uncertainty and easier phase discrimination than for*d*_*LV*/*RV*_, in particular when compared to RTG_GLOBAL_ and RTG_LOCAL_. Finally, in Figure 5D BA scatterplots for PCA descriptors of spatial complexity are presented. As for Pin Group, the LoA width is larger compared with the other BSPM parameters. Furthermore, point distribution is extremely scattered and BSPM measures associated with distinct intervention steps are difficult to distinguish, with most of z_D_ values far from zero, hinting at a weaker relation with epicardial RT dispersion, regardless of the metric considered.

## Discussion

In this study, we have compared state-of-the-art and novel parameters for the non-invasive assessment of the degree of cardiac repolarization dispersion from BSPMs. The use of an *ex vivo* torso tank model allowed us to test all metrics in a controlled environment and get rid of confounding factors that are typically encountered *in vivo*, e.g., respiration movement, use of anesthetics and lack of control on the mechanisms underlying a certain pathology. Importantly, this model helped us to reproduce global and local repolarization abnormalities due to purely electrical phenomena, which is crucial to understand electrical disorders which may affect structurally normal hearts. In addition, simultaneous invasive mapping of repolarization has enabled a better understanding of the relation between drug-induced changes in repolarization at the level of the tissue and manifestations of these phenomena on body surface as reflected on T-wave characteristics.

### T-Wave Duration and Dispersion of Repolarization

As in previous studies, we usually observed a prolongation of the duration of the T_PEAK_-T_END_ interval when repolarization gradients were created by drug perfusion. Changes in this index have been thought to reflect alterations in transmural dispersion of repolarization (Yan and Antzelevitch, 1998), and be prognostic of arrhythmic risk under a variety of conditions (Antzelevitch et al., 2007). Nevertheless, the ability of T_PEAK_-T_END_ interval to provide an absolute measure of transmural dispersion *in vivo* is still under debate (Xia et al., 2005), and other studies have supported the idea that this index may rather be a predictor of arrhythmogenesis as the result of increased global dispersion of ventricular recovery (Opthof et al., 2007). Even though we could not fully clarify the precise mechanisms behind this electrocardiographic manifestation (we could not perform endocardial mapping), our findings show that a higher degree of both global and local repolarization heterogeneity subsequent to drug intervention reflects on the lengthening of the terminal part of T-wave on the torso tank. This behavior has also been validated by BA plot analysis, and showed strong consistency with similar formulations for epicardial RT dispersion. In only one case (experiment 3 from Pin Group) a shortening of this interval was measured from EGMs after pinacidil injection. This may be potentially explained by a delayed effect of the drug, which was actually visible only during drug washout (not shown). Concerning the QT interval, different behaviors have been observed depending on the experimental setup. The sole administration of pinacidil in Pin Group caused a shortening of the QT_C_ interval, in line with previous studies (Padrini et al., 1992; Extramiana and Antzelevitch, 2004) reporting this ECG feature as a consequence of higher transmural dispersion of repolarization. On the contrary, the use of dofetilide led to QT_C_ prolongation, which may be proarrhythmic (Barrett et al., 2001). Overall, increased epicardial dispersion of repolarization, both local and global, was not reflected by a consistent behavior of QT interval on body surface, thus confirming the lack of specificity of this index, which reflects both the depolarization and repolarization process, thus to be interpreted with caution.

### T-Wave Duration Variability and Dispersion of Repolarization

Exacerbation of repolarization gradients was responsible for enhanced beat-to-beat variability of T_PEAK_-T_END_ duration. This phenomenon has been previously related to increased arrhythmic risk both clinically and experimentally (Pueyo et al., 2011; Piccirillo et al., 2013). Results from computational studies have corroborated the critical role of I_Ks_ and I_Kr_ currents in the temporal modulation of repolarization reserve at the cellular level, both in physiological and pathological conditions (Pueyo et al., 2011). In Piccirillo et al. (2013), temporal myocardial repolarization lability was associated with increased risk for SCD and also potentially correlated with a downregulation of potassium currents, which is typical of patients with congestive heart failure. Importantly, as argued by the same authors, temporal instability of T_PEAK_-T_END_ duration may be indicative of the presence of tissue areas containing non-homogeneous refractory periods, and under favorable conditions (e.g., ischemia) the recovery of excitability may lag behind the completion of repolarization as well (Burton and Cobbe, 2001). Although the exact cellular mechanisms could not be elucidated by our investigation, in line with these studies, we also observed that a decline in temporal stability of T_PEAK_-T_END_ duration during drug perfusion precisely followed changes in RT dispersion on the sock and showed good agreement with the reference invasive metrics, and this phenomenon was observed in all experiments. On the contrary, regional electrical disparities were not accurately identified by the QTD index, whose capability of assessing repolarization heterogeneity has been questioned by previous studies as well (Malik et al., 2000; Prenner et al., 2016). Indeed, QT interval is insensitive to shortening of regional repolarization in the presence of prolonged repolarization elsewhere (Burton and Cobbe, 2001) and its variability is likely the result of the different projections of the heart vector on the body surface, rather than true dispersion of repolarization (Di Bernardo et al., 2000; Malik et al., 2000; Burton and Cobbe, 2001).

### T-Wave Morphology and Dispersion of Repolarization

Overall, abnormal dispersion of electrical recovery strongly affected the morphology of T-wave. In our dataset, a decline in T-wave symmetry was found to be associated with higher dispersion of repolarization. This is in contrast to results from simulations presented by Di Bernardo and Murray (2000), who found that T-wave became more symmetrical with increased apico-basal and transmural dispersion. However, other studies presented opposite findings (Van Huysduynen et al., 2005; Vicente et al., 2015), and this may be due to the use of a non-realistic torso-heart model, with too simple geometries, and the lack of granularity in the modulation of action potential initiation at different sites (Di Bernardo and Murray, 2000). In contrast, electrocardiographic manifestations reported by our investigation are closer to those presented by Vicente et al. (2015), although alterations in T-wave profile presented in that study were provoked by other drugs acting on different ionic channels. In our experience, repolarization gradients led to a progressive loss of T-wave symmetry in all experiments, as a result of a steeper T-wave upslope and marked changes in the terminal portion of T-wave, both in terms of duration and area, with strong agreement with invasive measures of dispersion of repolarization. This effect was even clearer during experiments from Dof & Pin Group when both drugs were simultaneously injected, probably because differences between early and late repolarizing sites were more emphasized by the combined use of dofetilide and pinacidil rather than the injection of pinacidil alone and were easier to retrieve on body surface.

According to DTW, creation of repolarization gradients through pharmacological approach highlighted stronger differences in T-wave shape between left and right BSPM electrodes with respect to baseline. Morphological aberrations of T-wave profile as a consequence of repolarization inhomogeneity have been previously documented (Vicente et al., 2015; Ramirez et al., 2017), but mechanisms underlying such alterations are still unclear. Abnormal T-wave patterns have been observed during dofetilide-induced long QT syndrome (type 2) by Meijborg et al. (2015), who reported a correlation between increased interventricular dispersion of repolarization and appearance of notched waveforms on ECG, although shape assessment was rather qualitative. Alterations in ECG T-wave upslope have also been attributed to increased right-to-left RT dispersion from human hearts (Srinivasan et al., 2019). In Ramirez et al. (2017) DTW processing of T-wave accurately assessed risk for SCD in a population of congestive heart failure patients, although that retrospective study was more focused on temporal changes of T-wave rather than spatial aspects. Our research proposed a potential application of DTW processing as a descriptor of T-wave abnormalities in relation to dispersion of repolarization, and its capability of detecting variations in T-wave shape across leads. Interventricular differences in T-wave patterns were easier to appreciate in all experiments from Dof & Pin Group when both drugs were simultaneously injected, as for the *Asy* index. This finding is also confirmed by BA plot, which is characterized by narrower interval of agreement than in Pin Group and quasi-null z_D_ values, thus confirming good agreement with evidence from invasive recordings.

### T-Wave Complexity and Dispersion of Repolarization

PCA has proved to be a suitable tool for the description of cardiac activity from body surface, with prognostic value in SCD prediction (Porthan et al., 2013). Previous studies showed that T-wave non-dipolar components assessed by PCA are associated with increased dispersion of repolarization in a multitude of pathologies (De Ambroggi et al., 1997; Priori et al., 1997). Surprisingly, an inverse correlation between spatiotemporal organization of T-wave and repolarization uniformity was underlined by our analysis, albeit with some variability across the experiments. This unusual result is in line with (Zabel et al., 2000), showing that lower PCA ratios are predictive of post-myocardial infarction arrhythmic events, although with borderline statistical significance. The observed phenomenon may be due to an intrinsic limitation of our *ex vivo* model, as when we inject drug(s) in and outside the LAD artery we artificially create two distinct clusters with distinct recovery timings, with a steep transition at their border. This scenario is simpler than *in vivo* repolarizing tissue, where each myocardial cell differently contributes to the global behavior of the heart, therefore it may be more easily and accurately captured by low-rank PCA approximations rather than in the absence of drugs. Another potential explanation for this phenomenon may come from Van Oosterom’s T-wave model (Sassi and Mainardi, 2011; Laguna et al., 2016), which assumes surface T-wave genesis as the result of the counteraction between spatial distribution of RTs across the ventricular tissue and changes in the AP repolarization profile at the level of the single cardiomyocyte in a PCA-based framework. Indeed, according to Van Oosterom’s formulation, in substrates with increased heterogeneity of repolarization, at each time instant *t* surface T-wave *y*(*t*) can be decomposed into a weighted sum of a function *T*_d_ (also called the dominant T-wave) and its derivative ${\dot{T}}_{\text{d}}$:

$$\text{Y}\,\left(t\right)\approx {\text{w}}_{1}\,{\text{T}}_{d}+{\text{w}}_{2}\,{\dot{\text{T}}}_{d}\approx -\text{A}\,\Delta \,\rho \,\dot{d}\,\left(t-\bar{\rho }\right)+\frac{1}{2}\,\Delta \,\rho^{2}\,\dot{\text{d}}\,\left(t-\bar{\rho }\right)$$

With the weights **w**_1_ = −**A**Δρ and ${\text{w}}_{2}=\frac{1}{2}\,\Delta \,\rho^{2}$ dependent on RT dispersion Δρ across the tissue, *A* a transfer matrix, $d\,\left(t-\bar{\rho }\right)$ describing the repolarization phase of the transmembrane potentials of the myocytes of a given region, and $\bar{\rho }$ equal to the spatial average of local RTs over that region. As illustrated in Laguna et al. (2016), the unknown **T**_d_ and ${\dot{\text{T}}}_{\text{d}}$ functions can be estimated by SVD of the input BSPM matrix **Y** as:

$${\text{T}}_{d}=c_{1}\,\sigma_{1}\,{\text{v}}_{1}^{T},{\text{w}}_{1}=\frac{u_{1}}{c_{1}}$$

$${\dot{\text{T}}}_{d}=c_{2}\,\sigma_{2}\,{\text{v}}_{2}^{T},{\text{w}}_{2}=\frac{u_{2}}{c_{2}}$$

Where *c*_*1*_ and *c*_*2*_ are scalar constants which can be computed under suitable constraints (Sassi and Mainardi, 2011). This theoretical framework clarifies the simultaneous dependence of the second singular value σ_*2*_ on RT dispersion Δρ and on the AP downstroke velocity *d̈* $\left(t-\bar{\rho }\right)$, which may explain (at least in part) the unexpected trend of PCA features during drug injection. Specifically, in Pin Group the increased spatial heterogeneity of repolarization Δρwith progressive infusion of pinacidil is accompanied by K_ATP_ channel opening, causing shorter APD and steeper downstroke *d̈* $\left(t-\bar{\rho }\right)$ (Fedorov et al., 2011). However, this action is not strong enough to induce a significant change in spatial complexity of T-wave, maybe because a relatively small area (the LAD coronary) is affected by this intervention, thus drug-induced changes in repolarization cannot be correctly captured by high-order PCA approximations, and in particular by the spatial direction **u**_2_. Conversely, in Dof & Pin Group, the injection of dofetilide in extra-LAD regions may have caused a more marked reduction in AP downslope velocity *d̈* $\left(t-\bar{\rho }\right)$ compared with RT spatial dispersion Δρ (Vicente et al., 2015), with a stronger effect likely due to the involvement of a higher number of ventricular sites, and partial restoration of the initial conditions during pinacidil injection. Furthermore, other factors may contribute to the complexity of myocardium electrical recovery *in vivo*, including autonomic modulation (Ghuran et al., 2000) or ischemia (Al-Zaiti et al., 2011). To our knowledge, this is the first application of PCA to T-wave analysis in an experimental model, as it has been so far performed only in clinical human patients, with comparisons with invasive measures from explanted hearts performed only indirectly (Zabel et al., 1995). Further investigation is needed to clarify this divergence between experimental and clinical findings.

### Limitations and Perspectives

The study was performed on a limited number of hearts, thus the proposed BSPM markers should be tested on a larger dataset to corroborate their descriptive accuracy. However, evolution of BSPM indices was overall consistent across experiments, and deviations from expected trends were linked to experimental conditions (e.g., delayed drug effect, tissue block etc.).

Paucity of data may also have affected the outcome of the BA analysis of signal features, and in particular with respect to the width of confidence intervals and the impossibility to perform a statistical comparison over such a low number of samples. Nevertheless, most of BSPM indices demonstrated good agreement with all indices of epicardial dispersion of repolarization, thus proving that physiologically relevant information about repolarization alterations can be inferred by body surface T-wave analysis at multiple observation scales.

Although the spatial resolution of the signal processing framework presented in this study is not sufficiently high to clearly distinguish different mechanisms of recovery dispersion from body surface and/or localize tissue areas with abnormal timing, the proposed techniques can overall help to non-invasively detect repolarization abnormalities by exclusively exploiting the information provided by BSPMs, with no need for patient’s anatomy, requiring the use of more expensive imaging technologies. On the contrary, these tools could be used to detect repolarization anomalies at a larger scale of observation and potentially complement other approaches (e.g., electrocardiographic imaging or contact mapping) to identify diseased ventricular sites with higher precision at a second stage.

Body surface markers for repolarization variability were compared and validated with global and regional measures of epicardial RT gradients. Although reliable, as they straightforwardly account for tissue local properties, these measures should be interpreted with caution. Indeed, the computation of RT-dependent metrics may be affected by experimental maneuvers (e.g., loss of sock electrode contact during LAD cannulation). Additionally, the examined parameters only allow us to describe what happens on the epicardium, thus any unexplained behavior of RT gradient measures may be potentially influenced by other phenomena, either endocardial or transmural. To address this question, in a subset of two hearts from Pin Group (Experiments #2 and #3) we recorded transmural potentials through 8 plunge needles (four electrodes, 3 mm spacing) and we measured the average of the epi-to-endo RT gradients over four needles (two in LAD region, two in the posterior interventricular artery area). Changes in transmural dispersion of repolarization over time were either limited (Experiment #2: Baseline 28 ± 25 ms vs. 1/2 Pin 24 ± 25 ms vs. 1/1 Pin 19 ± 22 ms, repeated-measures one-way ANOVA *p* = 0.05) or absent (Experiment #3: Baseline 20 ± 14 ms vs. 1/2 Pin 20 ± 14 ms vs. 1/1 Pin 20 ± 11 ms, *p* = 0.82). Transmural needle mapping has not been performed on the other hearts due not only this weak evidence, but also to its technical challenges, e.g., the risk for myocardial damage and potential onset of ischemia that could have compromised the accomplishment of the experiments. Furthermore, the low number of needle probes and their uneven positioning were likely responsible for the variability of these results across the experiments and the impossibility to give even a preliminary interpretation of the absence/presence of epi-endo dissociation. As a consequence, future works may rather envisage the inclusion of simultaneous endocardial mapping (e.g., with a basket catheter) to explore additional mechanisms beyond epicardial repolarization heterogeneity more systematically.

Future research may include the comparison with other invasive features (e.g., from intracardiac or optical mapping), to better elucidate the mechanisms underlying body surface manifestations of repolarization dispersion, as well as their direction. For the same purpose, the use of *in silico* models may help validating BSPM features and possibly link them to specific mechanisms at lower scale.

In both datasets, drug injection provoked marked repolarization gradients, both globally at the level of the whole organ and locally between neighboring, smaller sites, and all examined BSPM parameters exhibited a consistent relation with regional and global invasive RT dispersion gradients. A potential perspective of this research may envision the inclusion of models with different interventions (e.g., to create sharp local/global repolarization gradients with globally/locally homogeneous repolarization, as it partially happened during experiment #3 from Pin Group) to investigate whether and how these forms of electrical unevenness selectively affect body surface potentials.

As explained in section 2, in keeping with (Cabasson and Meste, 2008), a fixed-length search window has been used for all electrodes to detect T-wave offset. While the range of values chosen has allowed for the segmentation of the entire T-wave with no spurious truncation in most of the BSPM leads, in other electrodes the time occurrence of T-wave end may have been slightly overestimated, with some impact on T-wave duration parameters. To overcome this issue, incorrect T_OFF_ time locations were usually fixed after visual inspection when possible, and our analysis confirmed that shifted detections did not affect significantly differences in duration between distinct drug states and explanted hearts.

Measures of QT interval duration and dispersion have been adjusted for heart rate using Bazett’s equation, in spite of the lack of a clear consensus on the use of this formula for porcine models. To overcome this limitation, we also analyzed uncorrected values of QT duration and spatial variability, and we concluded that their time evolution was independent of heart rate, which is expected to be stable in explanted perfused hearts.

With regard to PCA assessment of T-wave, in this document we report a detailed analysis of only two PCA-derived indices, although we have investigated a larger number of PCA markers of T-wave from the state-of-the-art (e.g., the relative T-wave spatial residuum, T-wave planarity, total cosine R-to-T; Merri et al., 1989; Malik et al., 2000; Korhonen et al., 2009; Porthan et al., 2013), and evaluated their ability to measure repolarization heterogeneity in our database when using between one and three principal components. In line with the findings presented in section “2.3.4,” all indices were characterized by an unexpected inverse correlation between T-wave complexity and ventricular recovery dispersion, therefore we considered it appropriate to include a few of them in this report to avoid redundancy. Moreover, as stated above, to clarify whether and how changes in AP downslope and cell-to-cell coupling affect PCA eigenvalues’ extraction during drug injection, meaningful insights could be provided by computational models of cardiac repolarization at the single myocyte level, which may help exploring mechanisms at a lower scale than our epicardial sock (Laguna et al., 2016).

In this work propensity to ventricular arrhythmias was not systematically explored, as we mainly investigated how substrate-dependent repolarization abnormalities reflected on T-wave properties rather than those trigger-related. Preliminary research from our group on a similar dataset has shown that arrhythmia susceptibility is strongly influenced both by the trigger location and timing, but also by the properties of the underlying substrate (Bear et al., 2019b), and that in vulnerable hearts T-waves are longer, flatter and more asymmetric (Meo et al., 2020). However, the potential of electrocardiographic features as a tool for arrhythmogenesis assessment in this model deserves further investigation.

Finally, a systematic comparison with T-wave features from an equivalent 12-lead ECG could not be performed, as in most of the experiments electrodes with noisy/low voltage signals were located close to lead III and precordial leads (especially V5-V6) and removed. This may also be object of future investigation, for potential translation of the proposed T-wave analysis tools into clinical routine, where standard ECG is more commonly used.

## Conclusion

This study investigated performance of several indices for the non-invasive assessment of different mechanisms of dispersion of ventricular repolarization from BSPMs. Our findings confirm that pathological alterations of cardiac recovery can be studied from body surface, and that a more detailed assessment of such aberrations can be obtained only if we look at multiple properties of T-wave, including duration, morphology and spatiotemporal organization. This research opens promising perspectives in the characterization of repolarization abnormalities, with potential applicability to clinical electrocardiology.

## Data Availability Statement

The raw data supporting the conclusions of this article will be made available by the authors, upon reasonable request. Requests to access the datasets should be directed to laura.bear@ihu-liryc.fr. A subset of the experimental data will be made available for the electrocardiographic imaging community, through the EDGAR project (http://www.ecg-imaging.org/), a collaborative effort by the Consortium for ECG Imaging.

## Ethics Statement

The animal study was reviewed and approved by the Directive 2010/63/EU of the European Parliament on the protection of animals used for scientific purposes and the local ethical committee.

## Author Contributions

MM conceived and designed the study, implemented the signal processing methods, analyzed and interpreted the results, and drafted the manuscript. PB equally contributed to the implementation of the study, methodological considerations, interpretation of results, and manuscript revision. LB and MC designed and performed the experiments, and provided insights into the interpretation of the results. EA also helped with experimental data acquisition. MH and OB provided feedback on the outcome of the study from a clinical and mechanistic point of view, respectively. RD helped with the conception of the study, provided feedback about the implementation of the methods and the interpretation of the results, and revised the manuscript. All authors have significantly contributed to this work.

## Conflict of Interest

MC was part-time employed by Philips Research. The remaining authors declare that the research was conducted in the absence of any commercial or financial relationships that could be construed as a potential conflict of interest.

## Abbreviations

APD: action potential duration
BA: Bland-Altman
BSPM: body surface potential map
CR: complexity of repolarization
Dof: dofetilide
DTW: dynamic time warping
ECG: electrocardiogram
EGM: electrogram
FIR: finite impulse response
LAD: left anterior descending
LoA: limit of agreement
LV: left ventricle
NMSE: normalized mean square error
Pin: pinacidil
PCA: principal component analysis
RT: repolarization time
RTG: repolarization time gradient
RV: right ventricle
SCD: sudden cardiac death.
